# Salmeterol and cytokines modulate inositol-phosphate signalling in Human airway smooth muscle cells via regulation at the receptor locus

**DOI:** 10.1186/1465-9921-8-68

**Published:** 2007-09-28

**Authors:** Natalie Smith, Claudia A Browning, Nathalie Duroudier, Ceri Stewart, Samantha Peel, Caroline Swan, Ian P Hall, Ian Sayers

**Affiliations:** 1 Division of Therapeutics & Molecular Medicine, University Hospital of Nottingham, Nottingham, UK

## Abstract

**Background:**

Airway hyper-responsiveness (AHR) is a key feature of asthma and a causal relationship between airway inflammation and AHR has been identified. The aim of the current study was to clarify the effect of proinflammatory cytokines and asthma medication on primary human airway smooth muscle (ASM) inositol phosphate (IPx) signalling and define the regulatory loci involved.

**Methods:**

Primary Human ASM cells were isolated from explants of trachealis muscle from individuals with no history of respiratory disease. The effect of cytokine or asthma medication on histamine or bradykinin induced IPx signalling was assessed by [^3^H] inositol incorporation. Quantitative Real Time PCR was used to measure mRNA levels of receptors and downstream signalling components. Transcriptional mechanisms were explored using a combination of 5'Rapid Amplification of cDNA Ends (5'RACE) and promoter-reporter techniques.

**Results:**

Treatment of Human ASM cells with IL-13, IFNγ or salmeterol for 24 hours lead to a modest augmentation of histamine induced IPx responses (144.3 +/- 9.3, 126.4 +/- 7.5 and 117.7 +/- 5.2%, p < 0.05). Similarly, TNFα, IFNγ or salmeterol treatment augmented bradykinin induced IPx responses (127.4 +/- 8.3, 128.0 +/- 8.4 and 111.7 +/- 5.0%, P < 0.05). No treatment significantly influenced sodium fluoride induced IPx responses suggesting regulation occurs at the receptor locus. Analyses of mRNA expression of components of the IPx pathway *i.e. *H1 Histamine Receptor (HRH1), B2 Bradykinin Receptor (BDKRB2), Gαq/11 and PLC-β1 identified that a significant induction of receptor mRNA (>2 fold) was a feature of these responses explaining the cytokine and spasmogen specificity. The HRH1 and BDKRB2 promoter regions were mapped in ASM and promoter-reporter analyses identified that salmeterol can induce HRH1 (>2 fold) and BDKRB2 (2–5 fold) transcription. The effect of cytokines on HRH1 and BDKRB2 promoter-reporter expression suggested a more complex regulation of mRNA expression involving additional loci to the core promoter.

**Conclusion:**

Our results indicate that the spasmogen specific receptor locus may be a key site of regulation determining the magnitude of spasmogen mediated ASM IPx responses during airway inflammation or following asthma medication. These data provide further insight into the molecular basis of AHR and extend our understanding of potentially detrimental effects associated with existing therapies used in the treatment of asthma.

## Background

Asthma is characterised by airway inflammation, reversible airway narrowing and airway hyper-responsiveness (AHR) caused by stimuli that normally elicit limited or no response. Excessive smooth muscle contractility has long been recognized as a feature of asthma, emphasised by the fact that drugs designed to prevent or reverse bronchoconstriction are still the most effective drug classes available. Spasmogens such as acetylcholine, bradykinin and histamine induce airway smooth muscle (ASM) contraction via inositol phosphate/calcium signalling mechanisms and thereby regulate the tone and calibre of the airways [1,2].

There is strong evidence for a causal relationship between airway inflammation and altered airway function including hyperreactivity to contractile agents and hyporeactivity to relaxant agents in asthma [3]. It has been shown that several cytokines are elevated in the lung during inflammation in asthma including; interleukin (IL)1β, Tumour Necrosis Factor (TNF)α [4] and IL13 [5]. Administration of the recombinant cytokine to mouse airways *e.g*. IL-13, has been shown to mimic several of the features of asthma including AHR [6]. Similarly, viral infection has been associated with asthma exacerbation and increased AHR and this is thought to be mediated at least in part by elevated interferon (IFN)γ expression in the airways [7]. Recently, it has also been shown that elevated CD4+IFNγ+ and CD8+IFNγ + cells are a feature of asthmatic airways and that CD8+IFNγ + cell numbers correlated with AHR [8]. A direct interaction between cytokines and Human ASM has been described involving alterations in the capacity of airway smooth muscle in culture to respond to relaxation and contractile agents [9,10]. Proinflammatory cytokines including; IL1β, TNFα and IL-13 have been shown to augment bradykinin induced Ca^2+ ^release in human ASM cells in culture [11-13]. Similarly, studies have been completed in order to determine the effect of cytokines on other agonist mediated responses (*e.g. *histamine or acetylcholine) including inositol phosphate (IPx) signalling, however, data have been conflicting [14,15]. Recently, a mechanism involving a role for the CD38/cyclic adenosine diphosphate pathway in regulating calcium homeostasis has been proposed at least in part to explain the effects of cytokines on agonist induced Ca^2+ ^signalling [16]. IL-13 treatment of Human ASM in culture was shown to increase expression of CD38 mRNA which was accompanied by an increase in ADP-ribosyl cyclase activity and net intracellular Ca^2+ ^responses to bradykinin and histamine [17].

Asthma maintenance medications including long acting β_2_-adrenoceptor agonists and inhaled corticosteroids have been shown to suppress AHR in the short term *in vivo *[18,19] and are the mainstay therapy for asthma. While clinically effective in many patients, concerns have been raised regarding potential adverse effects associated with chronic use of β_2_-adrenoceptor agonists [20-22] An increase in bronchial hyper-responsiveness in response to histamine has been observed following chronic use of the short acting β_2_-adrenoceptor agonist, salbutamol in asthma and COPD patients [23]. We have recently identified a potentially deleterious effect of prolonged exposure of Human ASM cells in culture to short acting β_2 _adrenoceptor agonists [24]. Prolonged treatment (24 hours) of Human ASM with salbutamol or isoprenaline augmented histamine mediated IPx production potentially providing a cellular mechanism explaining the clinical observations [24]. Recently, the outcomes of the Salmeterol Multicentre Asthma Research Trial (SMART) have been published which involved the assessment of 26,355 subjects [22]. This study indicated that prolonged use of the long acting β_2_-adrenoceptor agonist, salmeterol was associated with respiratory related deaths [22] suggesting that the adverse effects inferred for short acting β_2_-adrenoceptor agonists may also be of clinical importance for long acting β_2_-adrenoceptor agonist therapy.

Contractile agents including histamine, bradykinin and acetylcholine bind to G-protein coupled receptors (GPCR) on the surface of ASM which leads to the dissociation of Gαq from βγ subunits. Both Gαq and βγ subunits are capable of binding and activating phospholipase C (PLC)β1 which catalyses the generation of inositol 1,4,5-triphosphate (IP_3_) and diacylglycerol (DAG) from phosphatidyl 4,5-biphospate (IP_2_). These second messengers stimulate Ca^2+ ^release from intracellular stores and protein kinase C (PKC) activity, resulting in the activation of contractile and growth machinery in ASM [25].

There is accumulating evidence suggesting that the G-Protein Coupled Receptor (GPCR) may be a key regulatory locus *i.e*. the level of receptor expression provides specificity to the augmentation of signalling responses in airway smooth muscle cells [9]. This has been demonstrated for multiple GPCRs including; Cysteinyl leukotriene receptor 1 [26], β_2 _adrenoceptor [27], H1 histamine receptor [28] and B2 bradykinin receptor [29] where increases in mRNA levels are accompanied by augmented spasmogen responses following cytokines or drug treatment.

In the current study we aimed to comprehensively define the effect of key pro-inflammatory cytokines, IL1β, TNFα, IL-13, IFNγ together with dexamethasone and salmeterol on Human ASM IPx signalling pathways using a combination of cell signalling, gene expression and promoter analyses.

## Methods

### Reagents

All chemicals were analytical grade or higher. Plasticware was from Costar (High Wycombe, UK). All chemicals and reagents were purchased from Sigma-Aldrich (Poole, UK) unless otherwise stated. The [^3^H]-myo-inositol was purchased from Perkin Elmer (Bucks, UK). Inositol free DMEM was made to order by Invitrogen (Paisley, UK).

### Cell Culture

Human airway smooth muscle cells (ASM) were isolated from explants of trachealis muscle obtained from individuals with no history of respiratory disease as described [24,30]. Briefly, a section of trachealis muscle was isolated by dissection just above the carina and washed in Dulbecco's Modified Eagle's Medium (DMEM) supplemented with penicillin (200 U/ml), streptomycin (200 μg/l) and amphotericin B (0.5 μg/l). Explants (0.2 × 0.2 cm) were placed in 6 well plates and allowed to adhere. Cells were allowed to grow with fresh complete medium (10% foetal calf serum and 2 mM glutamine) added regularly, following 7–10 days growth cells approached confluency. As cells reached confluency, explants were removed and the cells were harvested using 5 mg/ml trypsin/2 mg/ml EDTA. Primary ASM cells exhibited >95% cell staining for α-actin. Airway smooth muscle cells were maintained in complete medium and passaged using trypsin/EDTA. Ethical approval for the use of primary cells was obtained from the local ethics committee. Airway smooth muscle cells from four individuals were used.

### Quantification of [^3^H]-inositol phosphates

[^3^H]-inositol phosphate formation in primary human ASM cells was quantified essentially as described [24,30]. Briefly, cells were plated in 24 wells at a density of 0.5 × 10^5 ^cells/well. Cells were allowed to grow for 2–3 days until ~80% confluent, then medium was replaced with 300 μl of inositol free DMEM, supplemented with 2 mM glutamine and containing [^3^H]-myo-inositol at a concentration of 8.1 MBq/ml. Cells were then incubated for 24 hours followed by the addition of IL-1β, TNFα, IFNγ (10 ng/ml), IL-13 (50 ng/ml) (PeproTech EC Ltd, London, UK), dexamethasone (1 μM) or salmeterol (1 μM) and incubated for a further 24 hours. The medium was then removed and cells were washed three times with 1 ml Hanks/20 mM HEPES and 300 μl Hanks/20 mM HEPES containing 20 mM LiCl was added. Cells were incubated for 30 min at 37°C, then agonists were added in a volume of 10 μl (100 μM Histamine, 0.1 μM Bradykinin or 10 mM Sodium Fluoride). Cells were incubated for a further 30 min and then the reaction was stopped by removing the medium and addition of 1 ml ice cold 50% methanol/60 mM HCl. Samples were stored at -20°C for at least 24 h. An 800 μl aliquot of each sample was neutralised using ~4.6 ml 5.5 mM Tris-HCl/11 mM NaOH. Total [^3^H]-inositol phosphates were separated from unincorporated [^3^H]-inositol using anion-exchange chromatography on Dowex-Cl columns and measured using scintillation counting [24,30].

### Quantification of HRH1, BDKRB2, GNA11 and PLCB1 gene expression

Human ASM cells were transferred to 100 mm Petri dishes at a density of 10^5 ^cells/ml. Cells were allowed to grow for 2–3 days until ~80% confluent. Complete medium was replaced with serum free medium and the cells were grown for a further 24 hours followed by the addition of cytokines or drugs (as described above). Isolation of cells for RNA extraction (at 4 and 24 hours post cytokine or drug addition) involved removing medium, a phosphate buffered saline (PBS) wash and collection in 1 ml PBS using a cell scraper. Cells were collected by centrifugation (300 × g 5 min), supernatant discarded and cell pellets resuspended in 50 μl PBS. 450 μl RNAlater was added to each sample followed by mixing and storage at -80°C. Total RNA was extracted from samples using the RNAeasy kit (Qiagen) as directed by the manufacturer. cDNA was generated from 1 μg RNA template using random hexamers and the Superscript kit (Invitrogen, Paisley, UK) as directed by the manufacturer. TaqMan assays specific for the Histamine H1 Receptor (*HRH1*), Bradykinin B2 Receptor (*BDKRB2*), Gαq/11 protein (*GNA11*) and phosholipase C-β1 (*PLCB1*) transcripts were designed using Primer Express Software (Applied Biosystems, Warrington, UK). See Table 1. Real Time PCR (Applied Biosystems) was carried out as directed by the manufacturer in a reaction volume of 20 μl containing ~100 ng of cDNA, 300 nM of each Primer, 200 nM Probe (Eurogentec Ltd, Southampton, UK) and 10 μl universal mastermix (Applied Biosystems). Differences in the quantity of cDNA template were normalised using a VIC labelled 18s ribosomal RNA endogenous control (Applied Biosystems). PCR was performed using the ABI-7700 and data collected using Sequence Detection Software (Applied Biosystems). Data was analysed as directed by the manufacturer and Ct values were normalised using the 18s ribosomal RNA signal. Relative differences in gene expression were calculated using the 2^dCt ^value as continuous variable (Sequence Detection System Compendium 7700 Version 4.0, Applied Biosystems).

**Table 1:** Primers and probes

	**REAL TIME PCR**			
**GENE**	**5'PRIMER**	**3'PRIMER**	**PROBE (5'FAM/3'TAMRA)**	**LOCATION**
HRH1	TCTCGGTGGCGGACTTGA	CATGAGCAGGTAGAGGATGTTCAT	CGTGGGTGCCGTCGT	ORF
BDKRB2	GATCAGCACCTTCCTGGATACG	GATGATGCGCTCGTCCTGG	CATCGCCTCGGCATCCTCTCCA	ORF
GNA11	TCAGCGAATACGACCAAGTCC	AGGGCTTTGCTCTCCTCCA	TGGAGTCGGACAACGAGAACCGG	Exon 5/6 boundry
PLCB1	CTGCCTGCTGTCTTTGTCTACATAG	TCGGATTGGGTTTGATAAAGCT	TGAAAGACTATGTGCCAGACACA TATGCAGATG	Exon 22/23 boundary

	**5'RACE PRIMER 1 (PCR 1)**	**PRIMER 2 (PCR 2)**		

BDKRB2	CACGAACAGCACCCAGAGGAAGG	CCAGCCCAGCCACTCCACTTG		

	**PLASMID CONSTRUCTION 5'PRIMER**	**3'PRIMER**	**LOCATION IN CONTIG**	**CONSTRUCT**

HRH1-1	CTGA**CTCGAG**CCTCCTCTCCCTGTGAGCTT	CTGA**AAGCTT**GCCGGCTCCGGGGAAAGTT	51,750 – 52,761	HRH1 1 kb
HRH1-2	CTGA**CTCGAG**GAAAGTGTGGACGCAGCCATT	CTGA**AAGCTT**GCCGGCTCCGGGGAAAGTT	50,782 – 52,761	HRH1 2 kb
HRH1-3	CTGA**CTCGAG**GTTCAATCATAGCTCACTGCAG	CTGA**AAGCTT**GCCGGCTCCGGGGAAAGTT	49,768 – 52,761	HRH1 3 kb
HRH1-4	CTGA**CTCGAG**TCCACAGCTTGTTGCACAGC	CTGA**AAGCTT**GCCGGCTCCGGGGAAAGTT	48,718 – 52,761	HRH1 4 kb
BDKRB2-1	CTGA**CTCGAG**CCCAGGCCACCCATAAACTG	CTGA**AAGCTT**CAAGCGGCATGGGCACTTCA	101,405 – 102,439	BDKRB2 1 kb
BDKRB2-2	CTGA**CTCGAG**GGGCGGATGTGTGGGTAGAT	CTGA**AAGCTT**CAAGCGGCATGGGCACTTCA	100,436 – 102,439	BDKRB2 2 kb
BDKRB2-3	CTGA**CTCGAG**CCTCCTATGTGCCGGGTGAT	CTGA**AAGCTT**CAAGCGGCATGGGCACTTCA	99,416 – 102,439	BDKRB2 3 kb
BDKRB2-4	CTGA**CTCGAG**ATGTTGGCCAGGCTGATCTC	CTGA**AAGCTT**CAAGCGGCATGGGCACTTCA	98,414 – 102,439	BDKRB2 4 kb

*HRH1 reference contig = AC083855, BDKRB2 reference contig = AL355102.5. Bold denotes HindIII and XhoI restriction sites.

### 5'Rapid Amplification of BDKRB2 cDNA ends

5'RACE was performed in order to identify the putative promoter regions of the *BDKRB2 *gene in Human ASM. Total RNA was extracted from Human ASM cells derived from two different donors. Total RNA extractions were performed using the RNeasy Mini Kit (Qiagen, Crawley, UK) as described above. 5'RACE was performed using the GeneRacer Kit (Invitrogen) according to the manufacturer's protocol. 2.5 μg of total RNA was used as template. The mRNA was reverse-transcribed with SuperScript II RT (Invitrogen) and the GeneRacer OligodT primer (50 μM) to generate RACE ready cDNA. A nested PCR approach was used to identify the 5' structure of the BDKRB2 gene; PCR1 (GeneRacer 5'Primer/BDKRB2 primer 1 specific for the open reading frame of the BDKRB2 gene). Reaction conditions included; 1 μl RACE ready cDNA template, 0.025 U/μl Platinum Taq DNA Polymerase High Fidelity (Invitrogen), 0.6 μM of each primer, 200 μM dNTPs, 2 mM Magnesium Sulphate and a staged PCR cycling approach; 94°C for 5 min, 94°C for 1 min 30 sec/72°C for 3 min × 5, 94°C for 1 min 30 sec/70°C for 1 min 30 sec/72°C for 3 min × 5, 94°C for 1 min 30 sec/68°C for 1 min 30 sec/72°C for 3 min × 20 and a final extension of 72°C for 10 min. The second stage PCR (PCR 2) utilised nested primers (GeneRacer 5' Nested Primer/BDKRB2 primer 2 specific for the open reading frame of the BDKRB2 gene). Reaction conditions were as described for PCR 1 except 1 μl cDNA (from PCR1) was used as template and PCR cycling involved; 94°C for 5 min, 94°C for 1 min 30 sec/68°C for 1 min 30 sec/72°C for 3 min × 25 and a final extension of 72°C for 10 min. 5'RACE products generated in PCR 2 were cloned into the pCR-4-TOPO vector using the TOPO TA Cloning Kit for Sequencing as directed by the manufacturer (Invitrogen). LB agar plates and LB broths (100 μg/mL ampicillin) were used to select and grow single colonies of positive clones. Plasmid DNA was purified using the FastPlasmid Mini Kit (Eppendorf, Cambridge, UK) and the presence of inserts was confirmed by restriction analysis with EcoRI (Promega, Southampton, UK). Positive clones showing an insert after restriction digest were sequenced using M13 primers (Invitrogen), ABI BigDye Terminator v3.1 Cycle Sequencing Kit and ABI Prism 310 DNA sequencer (Applied Biosystems). Sequences were analysed and the 5' structure of the *BDKRB2 *gene identified using Chromas v2.0 ([31]) and BLAST (NCBI).

### Luciferase Reporter Plasmid Construction

PCR primers were designed to generate 1, 2, 3 and 4 kb amplicons encompassing the predicted BDKRB2 transcription start sites (TSS) in HASM but not crossing the ExonI/IntronI interface (see Table 1). The most 3' TSS was at position 95,741,003 NCBI Build 35 (102,403 bp within AL355102.5)). A repeat polymorphism [GGTGGGGAC]n exists in this region therefore DNA fragments were generated to include the common n = 2 repeat observed in the Caucasian population [32]. Primers were designed to engineer a 5' HindIII and a 3' XhoI site into the DNA fragments to facilitate cloning into the pGL4-Luc2 vector (Promega). The BDKRB2 4 kb PCR included; 1 μl genomic DNA template (Caucasian), 0.025 U/μl Platinum Taq DNA Polymerase High Fidelity, 0.2 μM of each primer, 200 μM dNTPs, 2 mM Magnesium Sulphate and cycling parameters; 94°C for 2 min, 94°C for 30 sec/62°C 30 sec/68°C for 4 min × 30 and a final extension of 68°C for 7 min. The PCR product was cloned into the pCR-4-TOPO vector and sequenced (as described above). The sequenced pCR-4 vector containing the BDKRB2 4 kb fragment was used as PCR template for the generation of the BDKRB2 1, 2 and 3 kb fragments as described above. The 1, 2, 3 and 4 kb BDKRB2 fragments were excised from the pCR-4 vectors using HindIII/XhoI and cloned into pGL4-Luc2 using standard molecular biology techniques. An identical series of pGL4-Luc2 vectors containing 1, 2, 3, and 4 kb HRH1 promoter fragments encompassing all of the identified transcription start sites in Human ASM but not crossing the ExonI/IntronI interface (the most 3' TSS was at 52,754 bp within AC083855, [33]) were also generated (Table 1).

### Quantification of HRH1 and BDKRB2 core promoter transcriptional activity in CHO-K1 or Human ASM

CHO-K1 cells were cultured in complete DMEM (10% foetal calf serum and 2 mM glutamine) and harvested using 5 mg/ml trypsin/2 mg/ml EDTA. Cells were plated at a density of 25,000 cells/well into 48-well tissue culture plates (Corning, Sunderland, UK) using complete medium (see above) and allowed to grow for 24 h until ~80% confluent. Complete medium was replaced with serum free medium and the cells were transfected with 0.12 μg of pGL4-Luc2 using Fugene 6 (Roche) at a 3:1 ratio (Fugene:DNA). For derivatives of pGL4-Luc2 containing the HRH1, BDKRB2 or SV40 control inserts equimolar amounts of DNA were used and the amount of Fugene corrected accordingly. Cells were allowed to grow for 40 h prior to a PBS wash and harvested using Passive Lysis Buffer (PLB, 300 μl/well, rocked for 15 min and subjected to freeze/thaw at -80°C). Human ASM experiments were completed in an analogous manner except cells were allowed to grow for 24–72 hours until ~80% confluent. Following transfection cells were allowed to grow for 16 hours prior to the addition of cytokines or drug (at concentrations described above). Following 4 or 24 hours, ASM cells were washed with PBS and harvested using Passive Lysis Buffer as described in a volume of 100 μl/well. Firefly luciferase was quantified using 5 μl (CHO-K1) or 20 μl (ASM) of cell lysate and the luciferase assay system as directed by the manufacturer (Promega) and a Model TD-20e luminometer (Turner Biosystems, Sunnyvale, CA). Data was normalised to fold over the empty pGL4-Luc2 vector. Cytokine or drug treated samples were compared to medium alone treated samples by designated medium treated samples 100%.

### Promoter Database Analysis

Specific transcription factor binding sites were identified using; BioInformatics & Molecular Analysis Section [34], Transcription Element Search System [35], TFSEARCH [36] and WWW Signal Scan [37].

### Statistical Analyses

Differences between outcome measures for different treatment groups were compared by Analysis of Variance (ANOVA) in conjunction with Dunnett's Multiple Comparison Post Hoc Test or Students' T test as appropriate. Figures represent mean values (+/- S.E.M). Statistical analyses were completed using GraphPad Prism (GraphPad, San Diego, CA), a *P *value < 0.05 was considered significant.

## Results

### Exposure to Salmeterol, TNFα, IL-13 or IFNγ can augment the magnitude of histamine and/or bradykinin stimulated inositol phosphate signalling in Human ASM

In order to assess the effect of the micro-environment on subsequent IPx signalling responses in Human ASM we incubated cells with a series of pro-inflammatory cytokines implicated in the pathogenesis of asthma. These cytokines included; IL-1β, TNFα, IFNγ, or IL-13. The capacity of the ASM cells to produce IPx in response to two specific mediators of airway hyper-responsiveness; histamine and bradykinin and the non-specific Gαq/PLC activator sodium fluoride was determined. Similarly, the effect of two drug classes used routinely in the treatment of asthma was evaluated within the same model; *i.e. *the long acting β_2_-adrenoceptor agonist salmeterol and the steroid dexamethasone (Table 2). Significant differences were observed in the capacity of the ASM cells to produce IPx in response to histamine (p < 0.0001) or bradykinin (p = 0.0004) following cytokine or drug pre-treatment. There was not a statistically significant change in the capacity of the ASM cells to produce IPx following sodium fluoride stimulation (although differences were observed and there was a large degree of variation in these data). More specifically, IL-13, IFNγ and salmeterol were shown to significantly augment the capacity of the ASM cells to produce IPx in response to histamine stimulation (p < 0.05), however, these effects were modest; IL-13 (144.4 +/- 9.3%) compared to medium control (100%)), IFNγ (126.4 +/- 7.5%) and salmeterol (117.7 +/- 5.2%). Similarly, TNFα, IFNγ and salmeterol were shown to augment bradykinin mediated IPx production in ASM cells (p < 0.05), TNFα (127.4 +/- 8.3%), IFNγ (128.0 +/- 8.4%) and salmeterol (111.7 +/- 5.0%).

**Table 2:** Effect of pre-treatment with drugs or cytokines on the magnitude of Human ASM IPx signalling responses following histamine, bradykinin or sodium fluoride stimulation.

**Cytokine/drug**	**Histamine response**	**Bradykinin response**	**NaF response**
IL-1β	97.0 (+/- 4.1)	97.8 (+/- 4.3)	85.9 (+/- 8.1)
TNFα	113.0 (+/- 8.4)	127.4 (+/- 8.3) +**	102.8 (+/- 11.9)
IL-13	144.3 (+/- 9.3) +**	113.8 (+/- 7.0)	124.2 (+/- 15.6)
IFNγ	126.4 (+/- 7.5) **	128.0 (+/- 8.4) +**	132.3 (+/- 14.0)
Salmeterol	117.7 (+/- 5.2) **	111.7 (+/- 5.0) *	116.2 (+/- 10.9)
Dexamethasone	105.6 (+/- 4.4)	100.3 (+/- 5.0)	113.2 (+/- 10.4)

ASM cells were serum starved for 24 hours and then treated with medium alone, 10 ng/ml IL-1β, 10 ng/ml TNFα, 10 ng/ml IFNγ, 50 ng/ml IL-13, 1 μM dexamethasone or 1 μM salmeterol for 24 hours. Cells were loaded with [^3^H]-myo-inositol and IPx production following 100 μM Histamine, 0.1 μM bradykinin or 10 mM Sodium Fluoride stimulation was determined as described [30]. The IPx production in the medium alone treated cells was normalised to 100% and the other treatment groups determined relative to this. Data represents the mean response +/- S.E.M, n = 9 (three independent experiments for 3 donors). Data within groups was compared by ANOVA and post hoc Dunnett's Multiple Copmarison Test (+p < 0.05) or T-Test (*p < 0.05, **p < 0.01).

### Augmentation of IPx signalling by Salmeterol, TNFα, IL-13 or IFNγ is accompanied by elevated mRNA levels of the relevant GPCR

**Figure 1:**
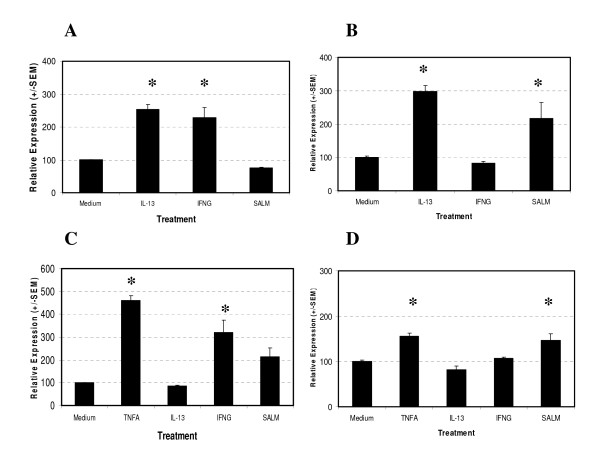
Effect of salmeterol or cytokine treatment on H1 Histamine Receptor and B2 Bradykinin Receptor mRNA expression in Human ASM. ASM cells were serum starved for 24 hours and then treated with medium alone, 10 ng/ml TNFα, 10 ng/ml IFNγ, 50 ng/ml IL-13 or 1 μM salmeterol for 4 or 24 hours. mRNA levels of the H1 Histamine Receptor and B2 Bradykinin Receptor were quantified using Real Time PCR and normalised using the 18s ribosomal RNA endogenous control. HRH1 mRNA quantification following 4 hours (A) and 24 hours (B) stimulation. BDKRB2 mRNA quantification following 4 hours (C) and 24 hours (D) stimulation. Data is normalised to medium control = 100%, n = 3 independent experiments in triplicate, mean +/- S.E.M. Dunnett's Multiple Comparison Test (*p < 0.05).

In order to test the hypothesis that the site of regulation determining the alteration in IPx signalling observed following cytokine or drug pre-treatment occurs at the receptor locus we determined the mRNA expression levels of the H1 Histamine and B2 Bradykinin Receptors in our samples using Real Time PCR, *i.e. *the two key receptors mediating histamine or bradykinin activation of human ASM cells ([29,38], Figure 1). Quantification of the HRH1 mRNA levels identified a significant induction of gene expression following drug or cytokine treatment for 4 (p < 0.0001) or 24 hours (p < 0.0001) (Figures 1A and 1B). A significantly elevated level of HRH1 mRNA expression was observed following 4 hours treatment of Human ASM with IL-13 (254 +/- 14.5% compared to medium control (100%)) or IFNγ(228.7 +/- 30.9%). At 24 hours the IL-13 treated cells maintained an elevated level of HRH1 mRNA expression (298.5 +/- 17.0%) and salmeterol treated cells demonstrated a significantly elevated level of HRH1 mRNA expression (217.3 +/- 47.1%). Quantification of BDKRB2 mRNA expression in Human ASM cells following cytokine or drug treatment identified a significant effect on receptor mRNA expression at 4 (p < 0.0001) and 24 hours (p < 0.0001) post treatment (Figures 1C and 1D). A significant induction of BDKRB2 mRNA expression was observed at 4 hours following TNFα (461.0 +/- 20.4%) or IFNγ (322.1 +/- 53.3%) treatment. Following 24 hours treatment of ASM cells an elevated level of BDKRB2 mRNA was observed in the TNFα (156.2 +/- 6.5) and salmeterol (147.1 +/- 14.7%) treated cells. Treatment of Human ASM cells with IL-13 did not significantly influence BDKRB2 mRNA expression.

### Augmentation of IPx signalling by Salmeterol, TNFα, IL-13 or IFNγ is not accompanied by elevated mRNA expression of the relevant G-protein or phoshopholipase C isoform

To further define the potential mechanisms responsible for the alterations in the capacity of Human ASM to generate inositol phosphates we quantified the level of Gαq/11 and PLCβ1 mRNA expression in these samples. The H1 histamine and B2 bradykinin receptors are thought to signal via coupling to the Gαq/11 and phospholipase Cβ1 isoforms [39-41]. Analysis of GNA11 and PLCB1 mRNA expression in Human ASM cells following 4 or 24 hours treatment with Salmeterol, TNFα, IL-13 or IFNγ did not identify any significant differences in mRNA levels compared to medium treated cells (data not shown). The absence of a significant alteration in GNA11 or PLCB1 expression levels does not exclude the potential that these molecules have altered activation states, which was not evaluated in the current analyses.

### Mapping and bioinformatics analyses of the HRH1 and BDKRB2 gene promoters in human ASM predicts multiple relevant transcription factor binding sites within the core promoter regions

**Figure 2:**
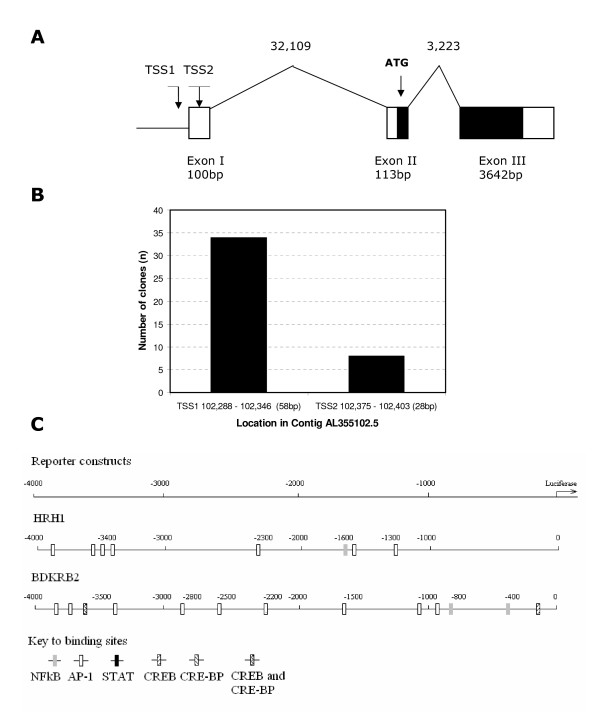
Identification of B2 Bradykinin Receptor gene transcription start site(s) and promoter region in Human ASM (A, B) and transcription factor analyses of core HRH1 and BDKRB2 promoter (C). Diagrammatic representation of the BDKRB2 genomic structure including identified exons and transcription start sites (TSS) in Human ASM (A) (Exon 1 5'boundary as described [42]). Number of pCR-4 clones clustering to the two potential transcription start sites (B). Data represents combined data from two Human ASM donors. Relevant transcription factor binding sites identified within the 4 kb of the core promoter of HRH1 and BDKRB2 genes by at least two of the four databases utilised (C).

In order to facilitate a more comprehensive analysis of the potential transcriptional mechanisms involved in HRH1 and BDKRB2 mRNA induction following Salmeterol, TNFα, IL-13 or IFNγ treatment the BDKRB2 transcription start site(s) and promoter regions were mapped in Human ASM cells using 5'RACE (Figure 2). Figure 2A shows a diagrammatic representation of the identified 5'region and transcript of the BDKRB2 gene in Human ASM. The BDKRB2 transcript was found to be composed of three exons separated by 32,109 bp and 3,223 bp introns respectively spanning ~39 kbps on chromosome 14. The coding region was found to be contained within exon 2 and 3 and two close transcription start sites were identified (Figure 2A and 2B). These data are in excellent agreement with previous analyses using a placental cDNA library [42]. The number of sequence clones representing the two potential transcription starts site are shown, with the dominant site (TSS1) being 5' to the previously reported TSS in placental cells ([42], Figure 2A and 2B). During the course of this study we also characterised the 5' region of the HRH1 gene in Human ASM which has now been reported elsewhere [33]. The HRH1 transcript was found to be predominantly (85%) composed of two exons; a 5' untranslated and an exon containing the coding region separated by a 104.4 kb intron [33]. The core HRH1 promoter was identified and three potential transcription start sites were identified [33]. 4 kb of each core promoter encompassing all of the identified transcription start sites was used as the basis for the generation of a series of promoter-reporter constructs. In order to potentially identify transcription factor binding sites with relevance to Salmeterol, TNFα, IL-13 or IFNγ treatment cell activation (*i.e. *NF-κB, AP-1, STAT, CREB and CRE-BP [43]) within these 4 kb sequences we completed analyses using transcription factor binding site databases (Figure 2C). Analysis of the HRH1 core promoter sequence using the TFSEARCH database identified the presence of AP-1 (6 sites, between 1–4 kb), NF-κB (1, 1–2 kb), CRE-BP (3, 1–4 kb), CREB (1, 3–4 kb), CRE/CRE-BP (1, 3–4 kb) and STATx (1, 3–4 kb) binding sites (data not shown) however, only the AP-1 (4 sites, between 1–4 kb) and NF-κB (1, 1–2 kb) were identified in two or more databases (Figure 2C). Analysis of the BDKRB2 core promoter using TFSEARCH identified NF-κB (6 sites, between 0–3 kb), AP-1 (5, 1–4 kb), STATx (2, 3–4 kb), CREB (3, 0–4 kb), CREB-BP (4, 0–4 kb) and CREB/CREB-BP (2, 0–4 kb) binding sites (data not shown). Following analyses using multiple databases NF-κB (2, 0–1 kb), CREB (2, 0–4 kb) and AP-1 (9, 0–4 kb) transcription binding sites were observed in two or more analyses (Figure 2C).

### Promoter-luciferase constructs encompassing 1, 2, 3 and 4 kb fragments of the HRH1 and BDKRB2 core promoters show transcriptional activity in CHO-K1 and Human ASM cells

**Figure 3:**
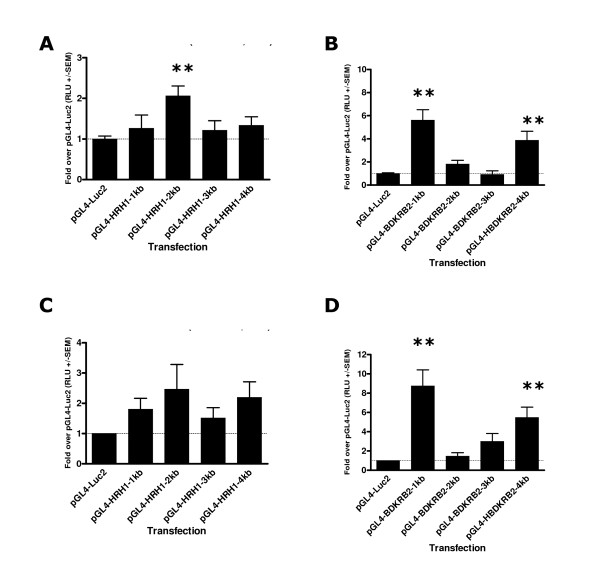
Transcriptional activity of *HRH1 *and *BDKRB2 *core promoter fragments. Luciferase activity in Human ASM cell lysates 20 hours post transfection with control, HRH1 and BDKRB2 luciferase constructs (A and B) (n = 4), Luciferase activity in Human ASM cell lysates 40 hours post transfection with control, HRH1 and BDKRB2 luciferase constructs (C and D) (n = 5). Dunnett's Multiple Comparison Test (compared to pGL4-Luc2 control **p < 0.01).

Preliminary luciferase experiments using CHO-K1 cells transfected with pGL4 constructs for 40 hours provided clear evidence that all of the HRH1 and BDKRB2 promoter-luciferase plasmids were transcriptionally active (all >15 fold over pGL4-Luc2 control, p = 0.0002 and p = 0.0004 respectively, data not shown). Interestingly the greatest activity in CHO-K1 cells was observed for the plasmids containing the longest segment of the two core promoters *i.e. *the HRH1-4 kb (106.5 +/- 34.3 fold over pGL4-Luc2, p < 0.01) and BDKRB2-4 kb (219.4 +/- 75.3 fold over pGL4-Luc2, p < 0.01) plasmids. Basal activity of HRH1 promoter containing plasmids transfected into Human ASM showed low level activity over pGL4-Luc2 at 20 hours post transfection with maximal activity observed for the pGL4-HRH1-2 kb transfection samples (2.1 +/- 0.2 fold, Figure 3A). Analyses of luciferase activity in Human ASM cells transfected with the series of BDKRB2 or control constructs for 20 hours demonstrated significant differences in activity between constructs (ANOVA, p = 0.0001). The BDKRB2 1 kb (5.6 +/- 0.9 fold) and 4 kb (3.8 +/- 0.8 fold) constructs showed clear activity (Figure 3B). The pGL4-BDKRB2-3 kb transfection samples showed similar to pGL4-Luc2 activity (Figure 3B). Transfection of Human ASM cells with HRH1 promoter containing constructs for 40 hours again demonstrated a modest transcriptional activity over pGL4-Luc2 as observed for the 20 hour transfections (maximum HRH1 2 kb, 2.5 +/- 0.8 fold, Figure 3C). Transfection of Human ASM cells with BDKRB2 promoter constructs again identified the 1 kb and 4 kb promoter constructs as demonstrating the highest level of activity as shown at 20 hours post transfection (8.7 +/- 1.7 fold and 5.5 +/- 1.1 fold respectively, Figure 3D). pGL4-SV40-Luc2 control luciferase levels were; CHO-K1 (649 +/- 99 fold) and Human ASM (20 hours 1229 +/- 173 fold, 40 hours 1255 +/- 157 fold). These data confirmed that the primary ASM cells were being efficiently transfected using this protocol.

### Stimulation of HRH1-luciferase transfected Human ASM for 4 and 24 hours with IL-13, IFNγ or salmeterol provides evidence for multiple mechanisms determining HRH1 mRNA levels

**Figure 4:**
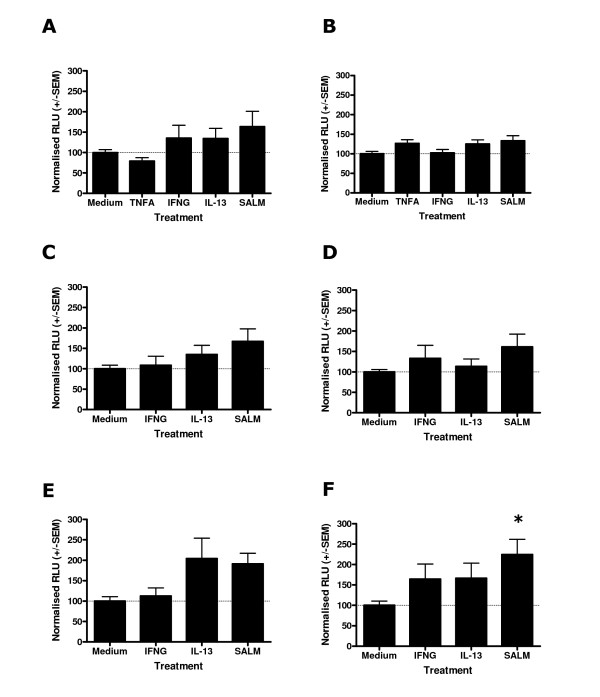
Effect of salmeterol or cytokine treatment for 4 hours on *HRH1 *promoter activity in Human ASM cells transfected with *HRH1*-Luciferase or control constructs. Complete medium was replaced with serum free medium and cells were transfected with 0.12 μg of pGL4-Luc2 using Fugene 6 (Roche) at a 3:1 ratio (Fugene:DNA). For derivatives of pGL4-Luc2 containing the HRH1 or SV40 control inserts equimolar amounts of DNA were used. Cells were allowed to grow for 16 hours prior to the addition of medium alone, 10 ng/ml TNFα, 10 ng/ml IFNγ, 50 ng/ml IL-13 or 1 μM salmeterol. Following 4 hours treatment cells were harvested and firefly luciferase was quantified. pGL4-Luc2 (A), pGL4-SV40-Luc2 (B), pGL4-HRH1-1 kb-Luc2 (C), pGL4-HRH1-2 kb-Luc2 (D), pGL4-HRH1-3 kb-Luc2 (E), pGL4-HRH1-4 kb-Luc2 (F) transfections. Data is normalised to the mean luciferase activity of each construct transfection treated with medium alone +/- S.E.M. (n = 4 independent experiments). Dunnett's Multiple Comparison Test (*p < 0.05).

**Figure 5:**
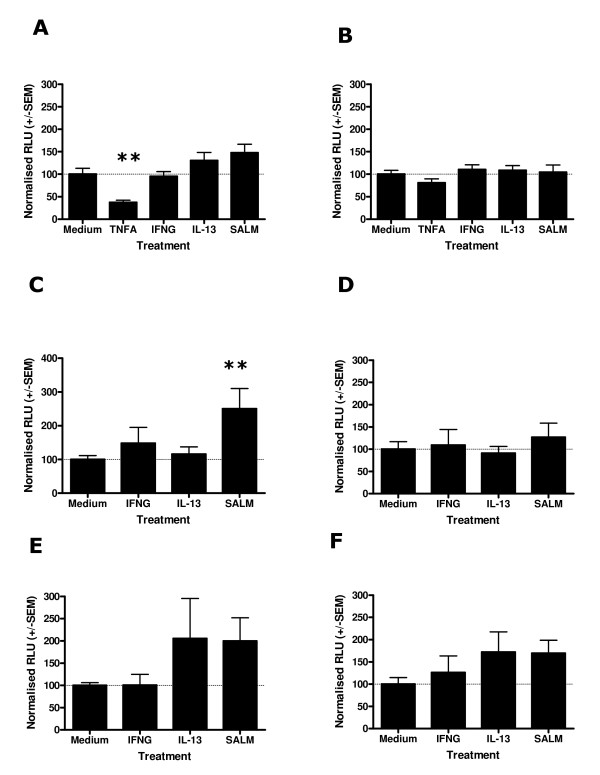
Effect of salmeterol or cytokine treatment for 24 hours on *HRH1 *promoter activity in Human ASM cells transfected with *HRH1*-Luciferase or control constructs. Human ASM cells were transfected and stimulated as described in Figure 4. Following 24 hours treatment cells were harvested and firefly luciferase was quantified. pGL4-Luc2 (A), pGL4-SV40-Luc2 (B), pGL4-HRH1-1 kb-Luc2 (C), pGL4-HRH1-2 kb-Luc2 (D), pGL4-HRH1-3 kb-Luc2 (E), pGL4-HRH1-4 kb-Luc2 (F) transfections. Data is normalised to the mean luciferase activity of each construct transfection treated with medium alone +/- S.E.M. (n = 5 independent experiments) Dunnett's Multiple Copmarison Test (**p < 0.01).

In order to investigate the effect of cytokines or salmeterol on HRH1 promoter activity in Human ASM, cells were transfected with the series of pGL4-HRH1 constructs for 16 hours and then stimulated with the appropriate cytokine or salmeterol for 4 (Figure 4) or 24 (Figure 5) hours prior to luciferase quantification. These analyses were not susceptible to transfection efficiency bias as comparisons were made within transfections. Predominantly, for these pGL4-Luc2 control transfections the background luciferase activity was not affected by stimulation with cytokines or salmeterol, however, a significant reduction in pGL4-Luc2 mediated luciferase activity was observed following 24 hours TNFα treatment (37.4 +/- 5.0 compared to medium control (100%), Figure 5A). Stimulation of Human ASM cells transfected with pGL4-SV40-Luc2 control plasmid did not identify any effect of TNFα, IFNγ, IL-13 or salmeterol treatment for 4 and 24 hours on SV40 mediated luciferase production (Figures 4B and 5B). Analyses of Human ASM cells transfected with the HRH1 1, 2, 3 and 4 kb promoter constructs and stimulated for 4 hours with IFNγ, IL-13 or salmeterol provided evidence that the level of transcription was influenced by treatment for the 3 and 4 kb constructs (ANOVA, p = 0.05 and p = 0.05 respectively Figures 4E and 4F). However, following post-hoc analyses only salmeterol significantly augmented transcription of the 4 kb core HRH1 promoter (224.5 +/- 37.4 versus medium control (100%), p < 0.05 Figure 4F). There was suggestive evidence that IL-13 may influence transcription of the HRH1 3 kb promoter (204.1 +/- 50.1) and IFNγ may influence the 4 kb construct (164.4 +/- 36.8, Figures 4E and 4F) although these differences were not statistically significant. Analyses of Human ASM cells transfected with the HRH1 1, 2, 3 and 4 kb promoter constructs and stimulated for 24 hours with IFNγ, IL-13 or salmeterol provided evidence that the level of transcription was affected for the 1 kb constructs (ANOVA, p = 0.03, Figure 5C) and that salmeterol significantly augments the HRH1 1 kb construct transcriptional activity (250.3 +/- 60.3, p < 0.05, Figure 5C). As previously, there was evidence that IL-13 influences transcription of the HRH1 3 kb promoter (205.3 +/- 90.2, Figure 5E).

### Stimulation of BDKRB2-luciferase transfected Human ASM for 4 and 24 hours with TNFα and IFNγ does not identify a significant effect on BDKRB2 transcription however, salmeterol stimulates BDKRB2 transcription

**Figure 6:**
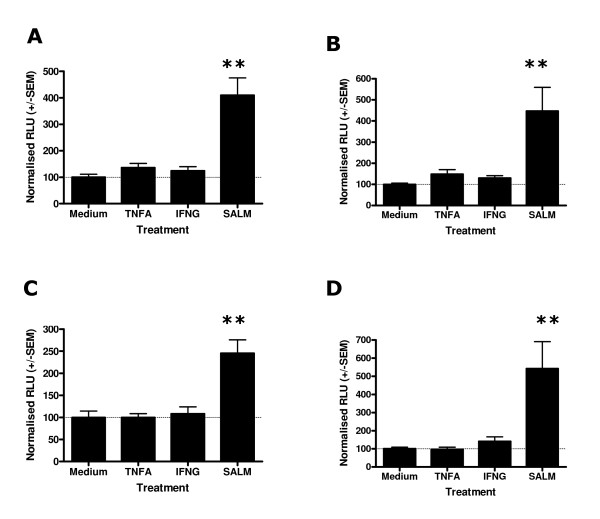
Effect of salmeterol or cytokine treatment for 4 hours on *BDKRB2 *promoter activity in Human ASM cells transfected with *BDKRB2*-Luciferase constructs. Human ASM cells were transfected and stimulated as described in Figure 4 except derivatives of pGL4-Luc2 containing the BDKRB2 inserts were used. Following 4 hours treatment cells were harvested and firefly luciferase was quantified. pGL4-BDKRB2-1kb-Luc2 (A), pGL4-BDKRB2-2kb-Luc2 (B), pGL4-BDKRB2-3kb-Luc2 (C), pGL4-BDKRB2-4kb-Luc2 (D) transfections. Data is normalised to the mean luciferase activity of each construct transfection treated with medium alone +/- S.E.M. (n = 4 independent experiments). Dunnett's Multiple Copmarison Test (**p < 0.01).

**Figure 7:**
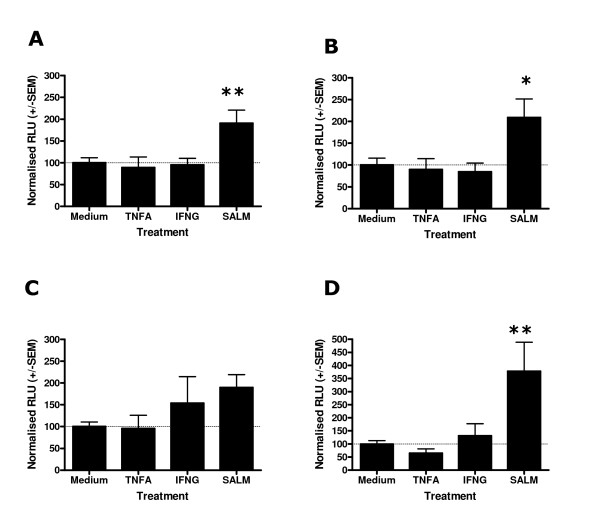
Effect of salmeterol or cytokine treatment for 24 hours on *BDKRB2 *promoter activity in Human ASM cells transfected with *BDKRB2*-Luciferase constructs. Human ASM cells were transfected and stimulated as described in Figure 4. Following 24 hours treatment cells were harvested and firefly luciferase was quantified. pGL4-BDKRB2-1kb-Luc2 (A), pGL4-BDKRB2-2kb-Luc2 (B), pGL4-BDKRB2-3kb-Luc2 (C), pGL4-BDKRB2-4kb-Luc2 (D) transfections. Data is normalised to the mean luciferase activity of each construct transfection treated with medium alone +/- S.E.M. (n = 5 independent experiments). Dunnett's Multiple Comparison Test (*p < 0.05, **p < 0.01).

In an analogous manner to that described for the HRH1 promoter analyses the effect of TNFα, IFNγ or salmeterol on BDKRB2 mediated transcription was evaluated using the pGL4-BDKRB2-luciferase transfected Human ASM cells. Following 4 hours stimulation with TNFα or IFNγ there were no apparent effects on BDKRB2 mediated transcription for the 1, 2, 3 and 4 kb constructs. Salmeterol treatment resulted in an augmentation of BDKRB2 mediated transcription for the 1, 2, 3 and 4 kb constructs (409.4 +/- 65.7, 446.7 +/- 111.9, 245.3 +/- 30.8 and 542.4 +/- 148.5 respectively, p < 0.01, Figure 6). Following 24 hours stimulation of BDKRB2-luciferase transfected Human ASM cells with TNFα, IFNγ or salmeterol a similar pattern to the 4 hour experiments was observed. TNFα and IFNγ stimulation did not significantly influence BDKRB2 mediated transcription (Figure 7). Salmeterol treatment of BDKRB2-luciferase transfected Human ASM cells for 24 hours significantly augmented BDKRB2 mediated transcription for the 1, 2 and 4 kb constructs (190.9 +/- 30.0, 208.8 +/- 42.7 and 377.7 +/- 110.7 respectively, p < 0.05, Figures 7A, 7B, 7D), however, the effect was apparent for all constructs.

## Discussion

In this study we aimed to clarify the effect of pro-inflammatory cytokines (IL-1β, TNFα, IFNγ, or IL-13) and two representatives of drug classes, dexamethasone and salmeterol on agonist mediated IPx signalling in Human ASM. To this end we have demonstrated that TNFα, IFNγ or salmeterol can significantly augment bradykinin induced IPx signalling responses and IL-13, IFNγ or salmeterol can significantly augment histamine induced IPx signalling responses in Human ASM. Further analyses using non-specific activation of the IPx pathway suggested a regulatory mechanism involving the receptor locus, which was confirmed by the quantification of the H1 Histamine and B2 Bradykinin receptor mRNA levels in treated cells. Finally, we sought to identify transcriptional mechanisms underlying these effects by mapping the core HRH1 and BDKRB2 promoter regions in Human ASM and completing promoter-reporter analyses. These data confirmed that salmeterol can induce HRH1 and BDKRB2 transcription, however, the cytokine analyses suggested a role for additional mechanisms regulating cytokine induced GPCR mRNA levels.

The mechanisms underlying the potentially deleterious effects of prolonged use of β_2_-adrenoceptor agonists in asthma are unclear at this time. This study represents the first demonstration that salmeterol can modestly augment bradykinin induced IPx signalling in Human ASM and has identified a mechanism involving the induction of B2 Bradykinin receptor mRNA via increased transcription at the core promoter. The promoter-reporter analyses showed a robust and pronounced effect of salmeterol on BDKRB2 promoter driven transcription which correlated with the identification of a consensus CREB site at -121 in the BDKRB2 promoter suggesting that all constructs (1–4 kb) should show induction of transcription. Interestingly, it has recently been shown that a CREB binding site at -44 to -51 in the rat BDKRB2 promoter is a major determinant of transcriptional regulation of the rat gene [44]. Salmeterol was also shown to augment histamine induced IPx signalling in Human ASM via an identical mechanism involving the H1 Histamine receptor, however, the reporter driven transcriptional affects were more modest potentially reflecting the low level of HRH1 promoter activity observed in the current study. These data extend our recent study that demonstrated that the short acting β_2 _adrenoceptor agonists; salbutamol and isoprenaline could augment histamine induced IPx signalling responses [24] and show that multiple contractile agent responses are affected by prolonged use of this class of drugs. In support of the current findings, previous data examining the effect of β_2 _adrenoceptor agonists on histamine mediated contraction in bovine ASM demonstrated a modest augmentation (1.5 fold) following fenoterol pre-treatment (18 hours) which was shown to be accompanied by an increase in HRH1 mRNA expression [28]. In this bovine system both transcriptional and post-transcriptional regulation were identified as determinants of HRH1 mRNA expression [28]. The clinical implications of these findings are that this mechanism may at least in part explain the accumulating evidence to suggest that prolonged use of long acting β_2 _adrenoceptor agonist monotherapy can lead to adverse effects in asthma as demonstrated in the SMART study [22]. This study of salmeterol use for 28 weeks was a multicentre, randomized, double blind, parallel group, placebo controlled design involving 26,355 subjects at 6,163 sites in the United States [22]. Interim analyses demonstrated that there was a significant increase in respiratory related deaths (24 vs. 11, RR 2.16 (95% CI 1.06 to 4.41)) and asthma related deaths (13 vs. 3, RR 4.37 (95% CI 1.25 to 15.34)) in the salmeterol versus placebo group [22].

Several studies have identified a direct interaction between cytokines and Human ASM leading to alterations in the capacity of airway smooth muscle in culture to respond to contractile agents [9,10]. TNFα can augment bradykinin induced Ca^2+ ^signalling (~2 fold) and IPx signalling (~2 fold) [12]. We have demonstrated similar affects and identified that induction of BDKRB2 mRNA is a feature of this response. Our data suggested that this induction of BDKRB2 mRNA may involve other mechanisms than transcription at the core promoter *e.g. *post-transcriptional mechanisms including RNA stability. The critical importance of 3'UTRs of genes determining mRNA expression has been established including a role for AU-rich elements (ARE) and RNA stabilising regions [45]. Several RNA binding proteins including; tristetraprolin (TTP), human antigen R (HuR) and AU-rich factor 1 (AUF1) have been identified that mediate RNA stability and therefore determine mRNA levels [46]. Interestingly, TNFα has been shown to induce HuR activity leading to increased eotaxin mRNA stability [47]. In support of the current study, TNFα has been shown to augment BDKRB2 mRNA expression in human embryonic lung fibroblast cells [48]. Nuclear run on assays suggested a prominent role for post-transcriptional mechanisms leading to elevated BDKRB2 mRNA following TNFα treatment [48] which would at least in part explain the lack of transcriptional regulation identified in the current promoter-reporter analyses. The 3'untranslated region of the Human B2 bradykinin receptor has been identified a key determinant of BDKRB2 mRNA expression [49]. TNFα has also been shown to augment bradykinin induced contractile responses in a mouse tracheal ring model and a role for increased B2 bradykinin receptor mRNA was identified [50] suggesting the mechanism exists across species.

IL-13 has been shown to augment histamine and bradykinin induced Ca^2+ ^signalling (~30% and 40% respectively) in Human ASM [13]. In the current study we have shown IL-13 specifically augments histamine induced IPx signalling and that this specificity involved selective regulation at the receptor locus. The modest induction of HRH1 transcription mediated by IL-13 in the promoter-reporter analyses again suggests multiple mechanisms determine the HRH1 mRNA level, *i.e. *a transcriptional mechanism does not adequately explain the robust >3 fold induction in mRNA levels observed. As part of a wider microarray study IL-13 has previously been shown to induce HRH1 expression in Human ASM (~2.6 fold, 4 hours) [51]. Interestingly, IL-4 has been shown to induce activity of the RNA stablilising protein HuR and increase mRNA expression levels of eotaxin in epithelial cells [47]. IL-4 and IL-13 can both activate the STAT6 pathway and therefore potentially the IL-13 dependent increase in HRH1 mRNA may involve post-transcriptional mechanisms involving HuR.

IFNγ has been shown to augment bradykinin induced Ca^2+ ^signalling (~1.5 fold) in Human ASM [52]. We have demonstrated that IFNγ can augment both histamine and bradykinin induced IPx signalling via induction of GPCR mRNA levels. Analysis of the BDKRB2 and HRH1 core promoter did not identify a transcriptional mechanism to explain the induction of mRNA levels in treated cells. Importantly, the β_1_-adrenergic receptor, a member of the GPCR family has been shown to be regulated via posttranscriptional mechanism which determine mRNA stability and level [53]. By inference, our core promoter analyses potentially suggest that post-transcriptional mechanisms may be important for GPCR mRNA regulation by cytokines.

**Figure 8:**
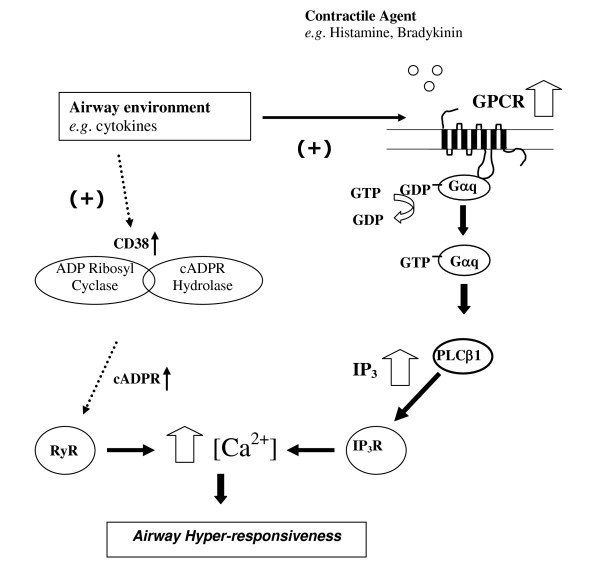
Schematic representation of mechanisms involved in the induction of hyperresponsiveness in Human ASM. Cytokines and salmeterol up-regulate mRNA expression of the relevant GPCR leading to increased IP_3 _production in response to specific contractile agents (as demonstrated in the current manuscript). In addition, cytokines can up-regulate CD38 leading to subsequent increased cADPR production [9]. Second messengers IP_3 _and cADPR activate the Ryanodine and IP_3 _receptor respectively leading to elevated calcium release from intracellular stores and downstream contractile responses.

In agreement with the proposed mechanism described in the current study, IFNγ has been shown to induce cysteinyl leukotriene receptor 1 (CysLTR1) mRNA expression in Human ASM with subsequent augmentation of LTD_4 _mediated Ca^2+ ^signalling responses [26]. These data suggest that regulation of Human ASM responses to multiple contractile agents involves regulation at the GPCR locus. In the current study we did not identify any effect of dexamethasone or IL1β on Human ASM IPx signalling in contrast to others [15,38], which may reflect cell donor/experimental differences. Previous data has demonstrated that *e.g. *IL-13 can augment KCl induced force generation in mouse tracheal rings suggesting a regulatory mechanism downstream of that examined in the current study [13]. A mechanism to explain these observations has been identified involving cytokine activation leading to elevated CD38 and subsequent cyclic ADP ribose (cADPr) which activates the ryanodine receptor (RyR) and modulates the calcium response in Human ASM [9]. Therefore, it is likely that regulation at the GPCR locus demonstrated in the current study provides some specificity and amplification of Ca^2+ ^responses via IP_3 _generation and that induction of CD38 also contributes to the overall magnitude of Ca^2+ ^and contractile responses (see Figure 8). Our finding that IL-13 did not influence bradykinin induced IPx production or BDKRB2 mRNA levels may suggest that augmentation of this specific agonist response is more dependent on the CD38/cADPr pathway although we can not exclude the influence of different cell donors/experimental designs.

These data demonstrate that a range of pro-inflammatory cytokines known to be over-expressed in the asthmatic lung alter Human ASM IPx signalling capacity and by inference may be involved in the molecular basis of AHR in asthma. It is important to note that TNFα identified in the current analyses as augmenting IPx production has also been shown to augment adenylyl cyclase activity in Human ASM [27]. Therefore, the potential contractile effects resulting from increased IPx production in response to cytokine and spasmogen may at least in part be countered by the increased relaxation capacity of ASM due to an augmentation of cyclic AMP production. It is interesting that IFNγ augmented responses to multiple contractile agents suggesting that this cytokine could have a key role in AHR development during *e.g*. viral infection and in asthma per se as CD8+IFNγ + cell numbers in the airways of asthma subjects correlate with AHR [8]. The finding that salmeterol can modestly augment Human ASM responses to contractile agents and potentially lead to AHR also has clinical implications. This study has identified some interesting findings however, the use of single dose and time points may have identified sub-optimal effects. Similarly, other limitations include the measurement of global IPx production as an outcome measure and not specific IPx production, cytosolic calcium and contractile responses which would have been desirable.

## Conclusion

We have presented evidence that the regulation of Human ASM responses to contractile agents can at least in part be explained by regulation at the GPCR locus. Several cytokines (TNFα, IFNγ, IL-13) and salmeterol modestly augment histamine and bradykinin IPx signalling responses in cultured Human ASM cells in a cytokine and agonist specific manner. This augmentation was accompanied by elevated mRNA levels of the specific GPCR providing a mechanism to explain this specificity. Finally, we demonstrated that cytokine modulation of HRH1 and BDKRB2 mRNA levels may involve additional mechanisms to a transcriptional mechanism involving the core HRH1 and BDKRB2 promoters. The effects observed for salmeterol mediated GPCR mRNA induction did involve transcriptional regulation at the core promoter. While the effects on IPx signalling were modest there is accumulating evidence that small changes in IPx signalling and subsequent cytosolic calcium concentrations can have physiological implications for contractile responses and airway hyper-responsiveness. These findings provide a greater insight into the molecular basis of AHR in asthma.

## Abbreviations

AHR, airway hyperresponsiveness, ASM, airway smooth muscle, PLC, phospholipase C, IPx, inositol phosphates, IL, interleukin, TNF, tumour necrosis factor, IFN, interferon, GPCR, G-protein coupled receptor, COPD, chronic obstructive pulmonary disease, DAG, diacylglycerol, IP_2, _inositol 4,5-biphosphate, IP_3_, inositol 1,4,5-triphosphate, PKC, protein kinase C, DMEM, Dulbecco's Modified Eagles Medium, EDTA, Ethylenediaminetetraacetic acid, PBS, phosphate buffered saline, Ct, threshold cycle, RACE, rapid amplification of cDNA ends, PCR, polymerase chain reaction, PLB, passive lysis buffer, NK-κB, nuclear factor κB, AP-1, activator protein 1, CREB, cyclic AMP response-element-binding protein, STAT, signal tranducer and activator of transcription, CHO, Chinese Hamster Ovarian.

## Competing interests

The author(s) declare that they have no competing interests.

## Authors' contributions

IS designed the study, carried out laboratory-based experiments (unless otherwise stated), completed data analyses and wrote the manuscript. NS completed the IPx signalling work and generated some of the RNA samples. CAB generated the promoter-luciferase constructs. ND and SP generated some of the RNA/cDNA samples. CES completed the promoter database analyses. CS designed the HRH1 TaqMan Assay. IPH contributed to the design of the study and writing of the manuscript. All authors read and approved the final manuscript.
